# Breast cancer screening: Impact on care pathways

**DOI:** 10.1002/cam4.2283

**Published:** 2019-06-06

**Authors:** Delphine Lefeuvre, Nathalie Catajar, Christine Le Bihan Benjamin, Norbert Ifrah, Frédéric De Bels, Jérôme Viguier, Philippe Jean Bousquet

**Affiliations:** ^1^ Health Data and Assessment Department, Survey, Data Science and Assessment Division Institut National du Cancer (French National Cancer Institute) Boulogne‐Billancourt France; ^2^ Screening Department, Public Health and Healthcare Division Institut National du Cancer (French National Cancer Institute) Boulogne‐Billancourt France; ^3^ Presidency Institut National du Cancer (French National Cancer Institute) Boulogne‐Billancourt France; ^4^ Public Health and Healthcare Division Institut National du Cancer (French National Cancer Institute) Boulogne‐Billancourt France; ^5^ Survey, Data Science and Assessment Division Institut National du Cancer (French National Cancer Institute) Boulogne‐Billancourt France; ^6^ Aix Marseille Univ, INSERM, IRD, Economics and Social Sciences Applied to Health & Analysis of Medical Information Marseille France

**Keywords:** breast cancer, care pathways, French cancer cohort, screening

## Abstract

**Background:**

Controversy persists concerning screening programs (SPs), related to a potential risk of overdiagnosis or the impact on survival. One of the main questions to be addressed concerns the aggressiveness of the related treatments.

**Methods:**

Using the "Cancer Cohort,” a national‐based cohort (medico‐administrative database), all women between the ages of 50 and 74 years and treated in 2014 for incident breast cancer were compared, according to whether their diagnosis was made following a mammogram performed within the framework of the SP (SP group) or outside it (NSP group).

**Results:**

A total of 23 788 women were identified: 13 530 (57%) in the SP group and 10 258 (43%) in the NSP group. The women in the SP group had a higher rate of in situ or localized invasive breast cancer. They had a higher rate of breast‐conserving surgery (82% vs 70%), and a lower rate of chemotherapy (34% vs 53%). These findings were observed irrespective of the stage. They had a higher rate of pathways involving breast‐conserving surgery followed by radiotherapy. Among women with metastatic cancer, those in the SP group had a lower proportion of liver, lung, brain, and bone metastases, and a higher proportion of lymph node metastases (other than axillary), irrespective of the time to onset of the metastases.

**Conclusion:**

The women in whom cancer was diagnosed following a mammogram performed in the context of the SP had less advanced cancer and less aggressive treatments. This observational study helps illustrate the benefit of the SP in France using a different approach.

## 1 INTRODUCTION

Breast cancer is the most common cancer in women worldwide.^1^ In France, the incidence of the disease was 54 062 new cases in 2015,^2^, ^3^ and it was the leading cause of cancer‐related mortality in women, with 11 913 deaths. The five‐year net survival of women diagnosed with breast cancer in France was 87% in 2005‐2010 (92% for women aged between 55 and 74 years).^2^, ^4^ Cancer screening programs have the potential to reduce mortality: in a SEER publication, the survival rate was 99% for breast cancer detected at a localized stage and 27% for metastatic cancer.^5^ The efficiency of screening in terms of mortality rates has been repeatedly challenged, however, since treatment has become more effective, primarily due to the diagnosis of lesions of questionable aggression—known as overdiagnosis—and the emergence of some cancers during the interval between two screenings.^6^, ^7^, ^8^, ^9^, ^10^, ^11^ A large number of studies have examined the impact of breast cancer diagnosis in these terms, but few have evaluated its impact in terms of the extent of treatment.^12^, ^13^, ^14^, ^15^ In France, the organized breast cancer screening program (SP) targets asymptomatic women aged between 50 and 74 years at average risk, that is, with no particular identified risk factors. They are systematically invited to undergo a clinical breast examination and a mammogram by an approved radiologist, free of charge, every 2 years. This program includes a systematic second reading of mammograms considered to be free from suspected abnormalities by a second expert radiologist. Initially offered in specific areas, the SP was extended to cover the whole of France in 2004. Following a rapid increase, the uptake rate stabilized at around 52% between 2008 and 2014, before falling slightly (50.7% in 2016).^16^ Consequently, the target uptake rate of 70% has never been attained.

Outside the SP, women may undergo what is known as an opportunistic screening mammogram, on individual prescription. This does not include double reading, and the interval between two mammograms is not defined. This type of screening would appear to apply to approximately an additional 10% women aged between 50 and 74 years, without documenting their level of breast cancer risk.^17^

The recommended treatments for breast cancer depend on the cancer stage. Briefly, in the case of in situ cancer, breast‐conserving surgery followed by radiotherapy sessions is recommended whenever feasible.^18^ In cases of invasive cancer, conservative surgery may be replaced by total mastectomy, and chest wall radiotherapy is recommended in the presence of poor prognostic factors or lymph node involvement. Chemotherapy is recommended, as a neoadjuvant regimen if the tumor is inoperable, and as an adjuvant regimen in the event of lymph node involvement, a metastatic situation, or poor prognostic factors.^19^

At a time when the organized breast cancer screening program is undergoing reform in France in order to improve its quality and uptake rate,^20^ the purpose of this study was to assess whether cancers diagnosed in the context of the SP are detected at an earlier stage or require less aggressive treatment than cancers diagnosed outside the SP (detection following an opportunistic mammogram or clinical symptoms).

## 2 METHODS

### 2.1 Data source

The French compulsory National Health Insurance system has compiled a database recording expenses related to hospitalization or outpatient care for every patient, called the French National healthcare data system (SNDS). The French Cancer Cohort^21^ has been extracted from this database and includes all individuals having suffered from cancer (in situ, invasive, or tumor with unpredictable outcome) since 2010, resulting in inpatient hospital care, outpatient care, or registration under the long‐term disease (LTD) status for cancer care that enables 100% cover by National Health Insurance. At the time of inclusion, no distinction was made between incident cases (recently diagnosed) and prevalent cases (diagnosed in previous years).

### 2.2 Selection of cases

All women aged between 50 and 74 years considered to be at average risk^22^ and having undergone a bilateral mammogram within or outside the SP prior to breast cancer diagnosis were included.

In order to limit the analysis to the incident breast cancer population in 2014, women suffering from breast cancer identified as a result of breast cancer‐related care during the 2010‐2013 period or a previous LTD for breast cancer were excluded. The concomitant presence of another cancer was further grounds for exclusion. In order to focus on women at average risk, who are the targets of the SP, women with a family history of breast cancer or dysplasia during the 2010‐2013 period, and liable to undergo specific individual follow‐up, were excluded. Similarly, women for whom no mammogram was found and those having undergone a unilateral mammogram were excluded.

### 2.3 Screening program mammogram (SP) vs mammogram outside the screening program (NSP)

Women having undergone a screening mammogram within the framework of the SP (SP group) or outside it (NSP group) were compared. In the NSP group, women could have had their mammogram in a context of opportunistic screening or in the event of clinical symptoms.

### 2.4 Diagnosis classification

Since the TNM (*Tumor, Node, Metastases*) stage is not recorded in the SNDS, the cancer stage was determined on the basis of the codes in the international classification of diseases, tenth version (ICD10) contained in hospital data in the year following the start of treatment.

The following stages are taken into consideration:

- in situ (Tis);
- localized breast cancer: early invasive breast cancer without regional lymph node involvement or remote metastases (All T,N0,M0);
- regional breast cancer: invasive with regional lymph node involvement (All T, N+, M0);
- metastatic breast cancer: invasive with onset of organ metastases or extraregional lymph node involvement during the year (All T, All N, M+). Three groups were taken into consideration according to the time to onset of metastases, since care pathways are liable to differ depending on the interval: less than 1 month postdiagnosis (synchronous), between 1 and 6 months, and over 6 months.

### 2.5 Statistics and data availability

The study being a population‐based study (ie involving all women in France fulfilling the inclusion criteria), no statistical test was performed, and differences were considered when “clinically” relevant.” To access data, contact the French national cancer institute (lesdonnees@institutcancer.fr).

## 3 RESULTS

### 3.1 Over 24 000 women included in the study

**Figure 1:**
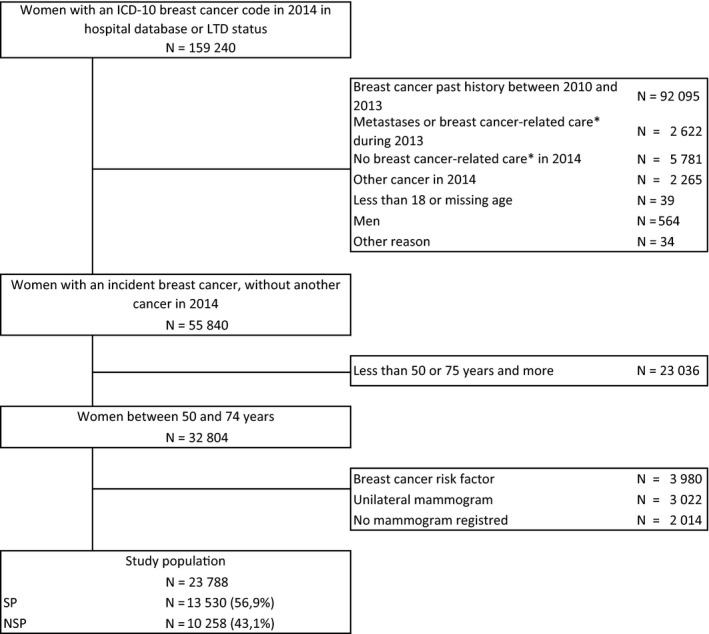
Flowchart. ICD10, The international classification of diseases—tenth version; LTD, long‐term disease; SP, screening program mammogram; NSP, mammogram outside screening program, as part of opportunistic screening or in the event of clinical symptoms. *Breast cancer‐related care, biopsy, breast surgery, chemotherapy, radiotherapy, palliative care

In 2014, 58 742 subjects had breast cancer identified as an incident (Figure 1). After excluding men, children, and individuals with another form of cancer, 55 840 women were taken into consideration, including 32 804 aged between 50 and 74 years. In the end, 13 530 women (57%) met the criteria for the SP group, and 10 258 (43%) met the criteria for the NSP group.

### 3.2 Women of similar age but with a slightly higher rate of metastatic involvement in the NSP group

The median age at diagnosis was similar in both groups: 62 [56‐67] years in the SP group vs 62 [55‐67] years in the NSP group. Localized breast cancers were more frequent in the SP group, whereas regional and metastatic breast cancers were more frequent in the NSP group (Table 1). Indeed, in the SP group, women had localized, regional, and metastatic breast cancers in, respectively, 75%, 15%, and 3% of cases, compared to 66%, 22%, and 9%, respectively, in the NSP group.

**Table 1:** Cancer stage by group

	Global repartition			Repartition without uncertain tumors and in situ breast cancer		
	SP + NSP (N = 23 788)	SP (N = 13 530)	NSP (N = 10 258)	SP + NSP (N = 22 574)	SP (N = 12 636)	NSP (N = 9938)
Tumor with uncertain behavior	138 (1%)	81 (1%)	57 (1%)			
In situ breast cancer	1076 (5%)	813 (6%)	263 (3%)			
Lobular (CLIS)	61 (6%)	39 (5%)	22 (8%)			
Canalar (CCIS)	692 (64%)	531 (65%)	161 (61%)			
Not precised	323 (30%)	243 (30%)	80 (30%)			
Local breast cancer	16 937 (71%)	10 155 (75%)	6782 (66%)	75%	80%	68%
Regional breast cancer	4269 (18%)	2054 (15%)	2215 (22%)	19%	16%	22%
Metastatic breast cancer	1368 (6%)	427 (3%)	941 (9%)	6%	3%	9%
M+ ≤1 month	498 (36%)	109 (26%)	389 (41%)	2%	1%	4%
M+ 1‐6 months	666 (49%)	245 (57%)	421 (45%)	3%	2%	4%
M+ >6 months	204 (15%)	73 (17%)	131 (14%)	1%	1%	1%

Among the women with metastatic cancer, those in the NSP group had a higher proportion of liver (28% vs 17%), lung (29% vs 25%), brain (14% vs 8%), and bone (52% vs 28%) metastases, and a lower proportion of lymph node metastases (other than axillary) than in the SP group (Table 2). Similar results were observed regardless of the time to onset of metastases.

**Table 2:** Localization of the metastases that appeared in the year since the diagnosis

	Among all metastatic women (M+)		Among women who had their first metastasis coded from the onset (M+ ≤1 month)	
	SP (N = 427)	NSP (N = 941)	SP (N = 109)	NSP (N = 389)
Liver	72 (17%)	262 (28%)	17 (16%)	134 (34%)
Lung	105 (25%)	276 (29%)	17 (16%)	121 (31%)
Brain	34 (8%)	134 (14%)	5 (5%)	69 (18%)
Bones	119 (28%)	493 (52%)	32 (29%)	251 (65%)
Other organ	95 (22%)	289 (31%)	25 (23%)	151 (39%)
Extraregional lymph node involvement	191 (45%)	340 (36%)	57 (52%)	129 (33%)

### 3.3 Higher rate of breast‐conserving surgery and lower rate of chemotherapy in the SP group

The women had a higher rate of breast‐conserving surgery in the SP group compared to the NSP group (82% vs 70%), and a lower rate of chemotherapy (34% vs 53%).

Among the women who underwent excision for in situ cancer, 22% had a total mastectomy in the SP group, and 27% in the NSP group (Table 3). These rates were 14% and 24%, respectively, for women with localized breast cancer, and 32% and 45% in cases of regional breast cancer.

**Table 3:** Treatments by cancer stage and group

Traitement	Ensemble des femmes		In situ		Local breast cancer		Regional breast cancer		Metastatic breast cancer	
	SP (n = 13 530)	NSP (n = 10 258)	SP (n = 813)	NSP (n = 263)	SP (n = 10 155)	NSP (n = 6782)	SP (n = 2054)	NSP (n = 2215)	SP (n = 109)	NSP (n = 389)
Breast surgery^a^	13 277 (98%)	9524 (93%)	809 (100%)	258 (98%)	10 011 (99%)	6543 (96%)	2041 (99%)	2188 (99%)	72 (66%)	109 (28%)
Conservative surgery	10 952 (82%)	6658 (70%)	627 (78%)	188 (73%)	8639 (86%)	4994 (76%)	1393 (68%)	1196 (55%)	46 (64%)	50 (46%)
Total mastectomy	2325 (18%)	2866 (30%)	182 (22%)	70 (27%)	1372 (14%)	1549 (24%)	648 (32%)	992 (45%)	26 (36%)	59 (54%)
Radiotherapy	11 813 (87%)	8583 (84%)	543 (67%)	152 (58%)	8998 (89%)	5745 (85%)	1942 (95%)	2089 (94%)	79 (72%)	219 (56%)
Chemotherapy^b^	4540 (34%)	5441 (53%)	3 (0%)	1 (0%)	2612 (26%)	2832 (42%)	1582 (77%)	1850 (84%)	83 (76%)	294 (76%)
Neoadjuvant	726 (5%)	1413 (14%)	1 (0%)		402 (4%)	751 (11%)	255 (12%)	477 (22%)	13 (12%)	43 (11%)
Adjuvant	4103 (30%)	4229 (41%)	2 (0%)	1 (0%)	2413 (24%)	2360 (35%)	1446 (70%)	1552 (70%)	49 (45%)	62 (16%)
Without surgery	105 (1%)	450 (4%)			20 (0%)	29 (1%)	11 (1%)	20 (1%)	24 (22%)	208 (53%)
Hormone therapy	9546 (71%)	7016 (68%)	53 (7%)	20 (8%)	7510 (74%)	4762 (70%)	1709 (83%)	1709 (77%)	79 (72%)	210 (54%)
Palliative care	54 (0%)	196 (2%)			5 (0%)	14 (0%)	6 (0%)	7 (0%)	11 (10%)	98 (25%)

a The partial and total mastectomy rates are calculated for those who had a breast surgery. In case of surgical revision with total mastectomy, total mastectomy is considered.

b It includes prescriptions for chemotherapy, targeted therapies, and immunotherapies. Women may have had neoadjuvant therapy, adjuvant, both, or treatment alone.

Chemotherapy was more frequent in cases of regional or metastatic breast cancer. It was used more frequently in the NSP group, particularly for the localized stage (42% of women in the NSP group and 26% in the SP group, respectively). This result applied to both neoadjuvant and adjuvant chemotherapies (Table 3). Conversely, at a similar stage, radiotherapy was used more frequently in the SP group, particularly for in situ cancers (67% of women in the SP group and 58% in the NSP group).

### 3.4 Care pathways

**Figure 2:**
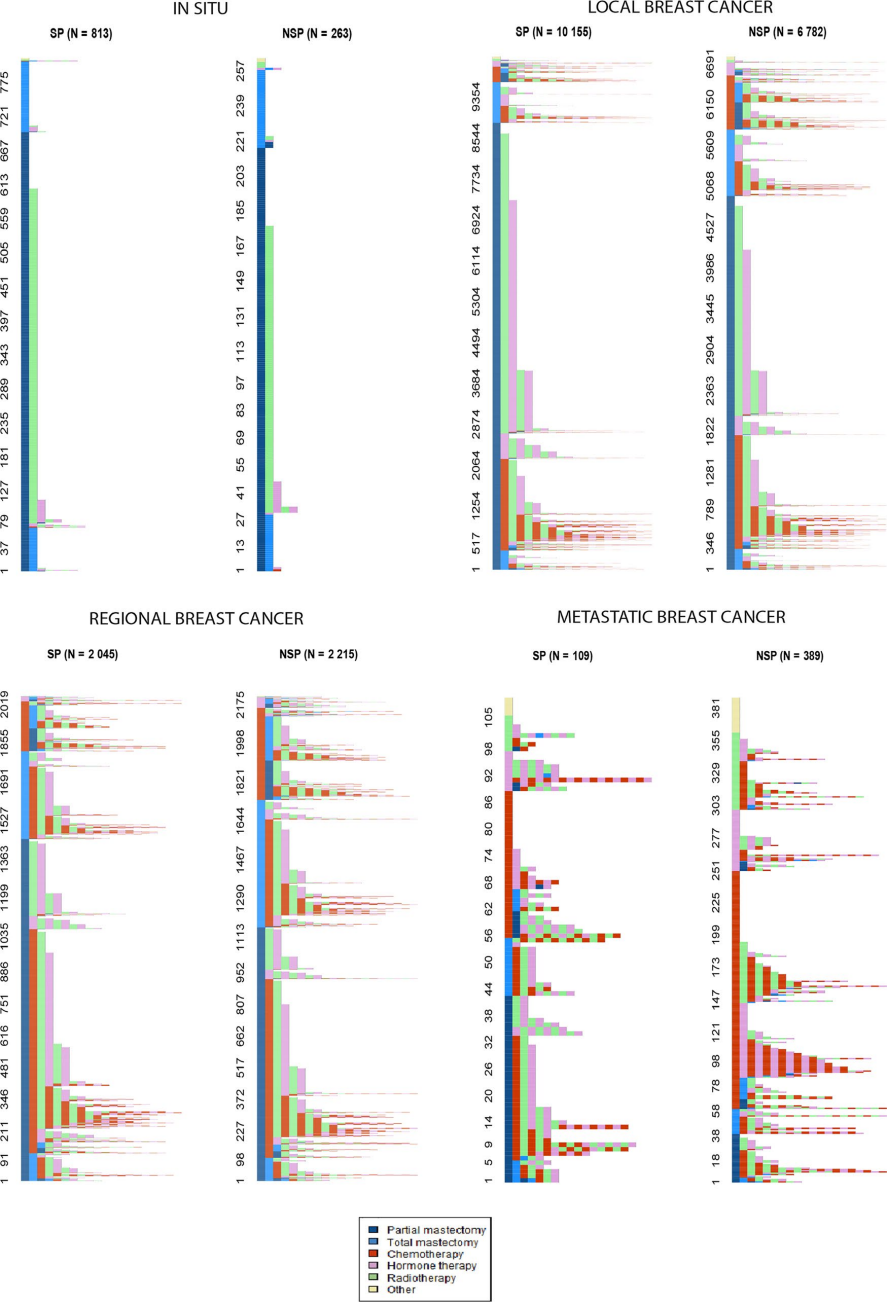
Care pathways by cancer stage and group. A color is attributed to each treatment: light blue for partial mastectomy, dark blue for total mastectomy, red for chemotherapy, etc Each care pathway is represented by a sequence of colored lines corresponding to the sequence of treatments received. For example, the care pathway of a woman treated by partial mastectomy followed by radiotherapy is represented by a strip of 2 colors: light blue and green. Alternating colors represented women having both radiotherapy or hormone therapy and chemotherapy during the same period of time. Strips of colors representing care pathways have a size proportional to the number of women following concerned pathways

Women with localized breast cancer had a higher rate of pathways involving breast‐conserving surgery followed by radiotherapy in the SP group than in the NSP group, which included more total mastectomies followed by adjuvant treatment, and more neoadjuvant chemotherapy (Figure 2,^23^). The findings are similar in cases of regional breast cancer. Finally, women with metastases had a higher rate of breast‐conserving surgery followed by adjuvant treatment in the SP group, whereas in the NSP group, almost half of the women's care pathways started with chemotherapy.

## 4 DISCUSSION

On the basis of the French Cancer Cohort data, 24 000 women having undergone a bilateral mammogram and diagnosed with breast cancer were identified in 2014. The women whose cancer was diagnosed following a mammogram carried out in the context of the SP had less advanced cancers and less aggressive treatments than the women who had undergone their mammogram outside the SP Indeed, in the SP group, women more often had in situ or localized cancer than in the NSP group (respectively, 6% and 75% compared to 3% and 66%), and fewer cancers with regional lymph node or metastatic invasion (respectively, 15% and 3% compared to 22% and 9%). Even when the cancer was metastatic, the metastases more often involved extraregional lymph nodes without visceral involvement in the SP group than in the NSP group. This study proposes findings based on real‐life observations rather than clinical trials that provide useful information with respect to the screening program's contribution to breast cancer control in women aged 50‐74 at average risk and without other concomitant cancer. We will be able to fine‐tune this work in the coming years, since the French National healthcare data system will soon make it possible to differentiate between women whose diagnosis was made following individual screening and those identified following clinical symptoms.

These results are consistent with previous studies conducted on the basis of regional or national registries. An analysis of six cancer registries made it possible to compare data before and after the introduction of a national SP in Bavaria in 2006:cancers were detected at an earlier stage after the introduction of the SP, resulting in a lower rate of total mastectomies (32.6% in 2000 vs 19.6% in 2008), a higher rate of radiotherapy (59.7% vs 66.6%) in parallel with an increase in breast‐conserving surgeries and a lower rate of chemotherapy (20.4% vs 13.1%).^15^ Similar results were obtained in the USA over the 1990‐2008 period, but in women aged 40‐49 years.^14^ Two retrospective studies comparing screening to clinical examination^12^, ^13^ suggested the same conclusions. However, these studies conducted on small sample sizes used a historical comparison, with different therapeutic options and some old data, or concerned age groups not consistent with those targeted in the SP Moreover, they could not differentiate between opportunistic screening and SP

More recently, a study realized in the Poitou‐Charentes region of western France between 2008 and 2009 and linking cancer registry, vital statistics, and French screening program data, also showed that screened detected cancers were diagnosed at a less advanced stage than interval cancers, themselves detected earlier than nonscreened‐detected cancers.^24^ Moreover, in this latter group, women had more advanced cancers, with metastatic and unresectable cancers, characterized by a greater proportion of palliative care.

Our study compares the SP and NSP groups over the same period, throughout the whole of France, using exhaustive data. Although there may be a lead time bias in the SP group, resulting in an overdiagnosis of in situ tumors*,* this study nonetheless demonstrates that the SP helps detect tumors at an earlier stage, requiring fewer total mastectomies and chemotherapy treatments. This is expected to result in a reduction in potential adverse effects and after‐effects, along with an improvement in patients' quality of life, on the one hand, and a reduction in treatment costs, on the other. Indeed, women who have had breast‐conserving surgery have a better body image than those who have had a total mastectomy,^25^, ^26^ as well as better physical fitness.^25^ Lymph node clearance, proposed in cases of axillary involvement, may result in pain, lymphoedema, and restricted movement, which may also impact women's quality of life.^27^, ^28^ Finally, in addition to the short‐ and long‐term side‐effects, chemotherapy would appear to affect some components of the SF‐36 scale (the Short Form [36] Health Survey, a standardized test for measuring quality of life) and result in more physical symptoms,^25^, ^27^, ^29^ which does not appear to be the case for hormone therapy and radiotherapy.^25^

The purpose of the study was to assess whether cancers diagnosed in the context of the SP required less aggressive treatment than those diagnosed outside the SP We therefore made a distinction between cancers treated within 6 months following a mammogram in the SP group and those treated more than 6 months after a mammogram in the NSP group. We assumed that, in the latter group, the diagnoses were not the direct consequence of the screening. This led to consider interval cancers occurring between 6 and 24 months after the initial screening in the NSP group.

Treatment costs increase with the stage at diagnosis. Subjects diagnosed at an early stage appear to be hospitalized for less time and to visit the emergency department less often, which tends to lower treatment costs.^30^ Moreover, according to a US study conducted on the SEER‐Medicare database, mastectomy associated with reconstruction would appear to increase the risk of complications and the cost of treatment compared to breast‐conserving surgery followed by radiotherapy sessions.^31^ Further complications, such as the presence of lymphoedema, may have an additional cost.^32^

Internal controls were performed to take into account coding errors in the SNDS, which was developed for economic and not epidemiological purposes. Some in situ cancers were classified as invasive cancer. Algorithms were constructed to approach clinical data, such as diagnosis stage. Furthermore, there has been a particular focus on only including data involving a low risk of problems, which explains the lack of data relating to lymph node clearance (confusion between sentinel lymph node and lymph node clearance). Information related to receptors, estrogen, progesterone, and human epidermal growth factor receptor 2 (HER2) was not available in the SNDS. Some studies reported a slightly lower proportion of luminal A and a relative higher proportion of HER2−/ER− in the NSP group. That over‐representation could increase the proportion of chemotherapy in the localized breast cancer subgroup. However, the treatment rates are actually in keeping with those found in the literature.^33^

The number of new breast cancer cases identified in the Cancer Cohort is similar to the 2015 national forecasts defined on the basis of cancer registries: the number of women aged between 50 and 74 years having had breast cancer was estimated to be 31 285,^2^, ^3^ vs 32 804 in this study. However, some of the cases identified may not be incident. Some women diagnosed outside a healthcare facility and whose treatment consists of hormone therapy may not have been identified, whereas others may have been included erroneously in the event of recurrence after a long time interval, due to a failure to reference the initial cancer. In order to limit this effect, women having received breast cancer‐related care in the course of the four previous years were not included in the study.

Estimates based on data from screening management structures find that 36 889 breast cancers were diagnosed over the 2013‐2014 period,^16^ that is, approximately 18 000 tumors detected by SP in the course of a year. In our study, 13 530 met the criteria for the SP group. A number of hypotheses may explain this difference. If the specific code for the reason for mammogram co‐payment exemption (free program) is missing, the examination is considered to fall within the scope of opportunistic screening and not the SP Furthermore, the maximum interval for attributing cancer diagnosis to a mammogram was set at 6 months between the mammogram and the first treatments. This potential bias results in a reduction in the disparities observed between the SP and NSP groups.

As TNM stage coding is missing in the SNDS data, the stages were defined on the basis of ICD10 codes. The proportion of in situ cancers is lower than that expected, some in situ cancers being classified as invasive cancer. Indeed, it is estimated to be 13.5% in the 50‐69 age group over the 2005‐2008 period in France.^34^ In our study, the rate of in situ cancers is approximately 5%, of which 64% DCIS, 6% lobular in situ cancer and 30% unspecified in situ cancers. Moreover, the proportion of cancers with regional lymph node involvement is lower than expected, whereas the proportion of localized breast cancers is higher. Indeed, based on national estimates of the breakdown by stages at diagnosis (excluding tumors with unpredictable outcome and in situ tumors, over the 2009‐2012 period), the rates of localized breast cancers, cancers with lymph node involvement and metastatic cancers in the 50‐74 age group are 65%, 28% and 7%, respectively (vs 75%, 19% and 6% in our study).^35^ Some women with lymph node involvement may have been included in the localized breast cancer group due to a coding error. However, while the cancer stage classification is incorrect for some women, these classification errors are, in principle, the same in the NSP and SP groups, and the differences between these groups persist.

Finally, among the women with metastatic cancer, those in the NSP group had a higher proportion of distant metastases. The main hypothesis should result in earlier diagnosis that could affect all patients in SP group, including metastatic patients. This was supported by the care pathways, with a higher rate (66% vs 28%) of breast surgery and a lower rate of palliative care (10% vs 25%) in the SP group.

## 5 CONCLUSION

The women whose cancer was diagnosed following a mammogram carried out within the framework of the SP had less advanced cancer and less aggressive treatments than the women diagnosed outside the SP This study, based on real‐life observations rather than clinical trials, helps illustrate the benefit of the SP in France.

The impact of regular screening on care pathways, and the detection of interval cancers (cancers diagnosed between two organized screening mammograms) will be examined in subsequent studies. Furthermore, the future enhancement of the French Cancer Cohort, with the addition of clinical data from oncology records, will enable these studies to be fine‐tuned. Finally, changes to mammogram coding will make it possible to differentiate between opportunistic screening and clinical symptoms in the NSP group.

## CONFLICT OF INTEREST

The authors declare that they have no conflict of interest.

## AUTHOR CONTRIBUTIONS

PJB and DL conceptualized and supervised the study; DL, CLBB, NC, and PJB designed the study (method); DL performed the analyses; DL, NC, and PJB wrote the original draft; DL, NC, CLBB, FdB, NI, JV, and PJB reviewed the article.
